# The contribution of energy systems during 30-second lower body Wingate anaerobic test in combat sports athletes: Intermittent versus single forms and gender comparison

**DOI:** 10.1371/journal.pone.0303888

**Published:** 2024-05-24

**Authors:** Erkan Tortu, Ibrahim Ouergui, Süleyman Ulupinar, Serhat Özbay, Cebrail Gençoğlu, Luca Paolo Ardigò

**Affiliations:** 1 Department of Coaching Education, Faculty of Sports Sciences, Trabzon University, Trabzon, Turkey; 2 High Institute of Sport and Physical Education of Kef, University of Jendouba, El Kef, Tunisia; 3 Research Unit: Sports Science, Health and Movement, University of Jendouba, El Kef, Tunisia; 4 Faculty of Sports Sciences, Erzurum Technical University, Erzurum, Turkey; 5 Department of Teacher Education, NLA University College, Oslo, Norway; University of Split, CROATIA

## Abstract

Combat sports, encompassing a range of activities from striking and grappling to mixed and weapon-based disciplines, have witnessed a surge in popularity worldwide. These sports are demanding, requiring athletes to harness energy from different metabolic pathways to perform short, high-intensity activities interspersed with periods of lower intensity. While it is established that the anaerobic alactic (ATP-PC) and anaerobic lactic systems are pivotal for high-intensity training sessions typical in combat sports, the precise contribution of these systems, particularly in varied training modalities such as single (SMT) and intermittent (IST) forms of the 30-second Wingate test, remains inadequately explored. This study aims at comparing performance outputs, physiological responses and gender differences during the SMT and IST forms of the 30-second Wingate test. Thirty-three highly trained combat sports athletes (17 women, 16 men; 10 boxing, 8 wrestling, 8 taekwondo and 7 karate) randomly performed SMT and IST. The IST consisted of three 10-second all-out attempts separated by 30 seconds of passive recovery, whereas the SMT was a single 30-second maximal effort. Resting, exercise and post-exercise oxygen uptake and peak blood lactate value were used to determine the metabolic energy demands via the PCr-LA-O_2_ method. The findings showed that total metabolic energy expenditure (TEE), ATP-PCr system contribution and the output of mechanical variables were higher in the IST than in the SMT form (all p<0.001). In contrast, the contribution of glycolytic and oxidative systems was higher in the SMT form (all p<0.001). However, exercise form and gender interaction were not significant (p>0.05). In combat sports, performance is not only determined by physiological and technical skills but also by metabolic energy input and efficiency. Therefore, our results can provide a comparison regarding the effects of exercise type and gender on metabolic energy metabolism to design the training of combat sports athletes.

## Introduction

Combat sports have been increasing in popularity, attracting a larger audience [1]. These sports, which are influenced by numerous physiological variables, encompass striking (e.g., boxing, karate and taekwondo), grappling (e.g., judo, Greco-Roman and freestyle wrestling), mixed (such as hapkido and mixed martial arts) and weapon-based (e.g., fencing and kendo) [1, 2]. To enhance the training and athletic performance of combat sports athletes, it is essential to understand the physiological responses associated with each sport [3]. Combat sports require the combination of different metabolic energy systems during training and fights [1, 4]. The activities require short bursts of high-intensity movement, interspersed by extended periods of lower-intensity activities [5]. The specific metabolic energy system contributions depend on the type of training being performed [1, 3]. For instance, during high-intensity training sessions such as sparring or pad work, the anaerobic energy systems are primarily used [6]. In particular, the anaerobic alactic (ATP-PC) system is activated during the initial few seconds of the activity, providing the immediate energy required for explosive movements such as throwing a punch or executing a takedown [7]. Afterwards, the anaerobic lactic system takes over, providing substantial metabolic energy for up to two minutes of high-intensity exercise [1, 8].

Sprint interval training (SIT) is a high-intensity exercise modality that can improve both aerobic and anaerobic fitness [9]. Evidence suggests that SIT induces diverse physiological adaptations, including an increase in skeletal muscle oxygenation, enhanced muscle oxidative capacity and peak oxygen absorption [10, 11]. Additionally, SIT has been associated with an increase in both anaerobic power and glycolytic enzyme activity [12]. As this type of training requires a much lower time commitment than traditional aerobic exercise, it is an attractive option for many populations, from the sedentary to the highly trained athlete [10]. Modified SIT protocols typically consist of 10–15 second work bouts with 2–4 min of rest, as opposed to the original SIT protocol. which involved 4–6 30-second all-out work bouts with 4 min of recovery [10].

The physiological differences between repeated and single exercise forms have yet to be fully elucidated. Previous studies have largely focused on comparing the energy provided by various metabolic pathways during various types and durations of maximal exercises, such as running, cycling and swimming [13–15]. However, the effect of repeated-sprint components on performance measures, physiological responses and metabolic energy expenditure has yet to be thoroughly investigated [16]. It is apparent that varying the design of a repeated-sprint exercise can produce distinct results [17]. Whereas in team sports training can be performed using protocols with standard rest intervals, protocols with a work-to-rest ratio are better suited for individual workouts [18]. Thus, further research is needed to determine how an athlete’s energy metabolism changes when he or she completes maximal repetitions of an exercise rather than a single all-out effort.

Research on the influences of gender on physiological and performance characteristics and gender-related variances and similarities in sports performance has recently attracted more attention [19, 20]. It is commonly known that men have stronger muscles than women and they produce more power [21]. However, studies have also shown that women are more resistant to fatigue than men [22]. Moreover, it has been demonstrated that men outperform women in repeated sprint tests [21, 23]. Furthermore, researches suggest that men and women have different energy metabolisms connected to exercise [1, 21]. Specifically, women, for instance, use less glycogen when exercising at the same intensity as males [24, 25]. Anaerobic-based energy systems are supposed to contribute to maximum performance during repeated sprints [26, 27]. Therefore, another factor contributing to this performance gap between men and women may be the more considerable contribution of anaerobic-based energy systems in males than in women during repeated sprints [28, 29].

Previous researches have largely focused on performance factors, fatigue, recovery mechanisms and gender differences during an exercise task [23, 30–32]. However, there is a dearth of data on gender differences in the contribution of metabolic energy systems between repeated and single forms of 30-second Wingate exercise bouts [21, 23]. Consequently, this study aims to compare performance, physiological responses and gender differences during the 30s and 3×10s Wingate exercise types and to ascertain the impact of gender on these procedures. We hypothesized that variations in the single all-out and repeated exercise designs and gender may affect the metabolic energy the system contributes overall or during rest periods.

## Materials and methods

### Participants

The sample size estimation was determined using the G*Power software (Version 3.1.9.4, University of Kiel, Kiel, Germany). Using the F test family (ANOVA: repeated measures and within-between factors) with α set at 0.05 and power (1-β) set at 0.80, the effect size f calculated was 0.257. The analysis revealed that 32 participants were required to approach an actual power of 81%.

The study enlisted highly trained combat sports athletes through a structured recruitment process that took place from February 3rd, 2023, until August 1st, 2023. Athletes were primarily recruited from national combat sports (boxing, wrestling, taekwondo and karate) teams and clubs, ensuring a high level of training and competitive experience. Our methodology, which consolidates athletes from various martial arts into a single group, aims to improve the applicability of our results across the spectrum of combat sports. This decision is based on the widespread popularity and the physical diversity—from striking to grappling—of these four martial arts, along with their status as both Olympic and non-Olympic disciplines. The inclusion criteria were as follows: (1) a minimum of seven years of training experience in combat sports; (2) active participation in official national competitions; and (3) aged between 18 to 30 years, to ensure participants were within the peak performance age range for combat sports. Exclusion criteria included (1) any musculoskeletal injury reported within six months prior to the study, which could affect performance in the Wingate test; (2) use of performance-enhancing drugs or any medical condition that could influence metabolic responses to high-intensity exercise; and (3) inability to comply with the study’s protocols, including the dietary restrictions before testing sessions.

A total of 33 highly trained combat athletes (17 women, 16 men; 10 boxing, 8 wrestling, 8 taekwondo and 7 karate) volunteered to participate in this study. The descriptive characteristics of participants are presented in Table 1. This study was conducted in accordance with the principles of the Declaration of Helsinki and approved by the Trabzon University Non-Interventional Clinical Research Ethics Board (Date: 08.10.2022; NO: E-81614018-2200045613). Written informed consent was obtained from all subjects involved in the study.

**Table 1:** Descriptive characteristics of men and women athletes.

Variables	Men (n = 16)	Women (n = 17)	p	d
Age (y)	21.56 ± 2.6	20.82 ± 2.3	**0.335**	0.30
Experience (y)	8.66 ± 1.2	7,50 ± 1.3	**0.910**	0.93
Height (cm)	180.3 ± 4.4	167.2 ±3.6	**0.000**	3.26
Body weight (kg)	75.18 ± 6.6	63.16 ± 7.5	**0.000**	1.70
Body fat (%)	14.11 ± 2.0	18.19 ± 4.4	**0.000**	1.19
Fat Mass (kg)	10.52 ± 6.1	11.40 ± 3.6	**0.070**	0.18
Fat Free Mass (kg)	64.66 ± 4.1	51.76 ± 4.5	**0.000**	2.99
Body Mass Index (kg/m^2^)	23.20 ± 3.3	22.63 ± 2.0	**0.740**	0.20
VO_2_ (L.min^-1^)	4.14 ± 0.4	3.40 ± 0.3	**0.000**	2.09
VO_2_(ml·kg·min^−1^)	55.20 ± 5.9	54.62 ± 5.1	**0.357**	0.10

Note. Values are means ± standard deviations.

### Experimental design

In this descriptive and repeated-measures study design, participants visited the Trabzon University of Sports Performance Analysis and Talent Center Laboratory three times, separated by 48–72 hours. Prior to the assessments, participants were familiarized with the testing tools and procedures and, after that, performed a graded exercise test to exhaustion to calculate the maximum oxygen consumption (VO_2max_). They performed SMT and IST (with 30-second rest breaks) in random order, and oxygen consumption (VO_2_) and heart rate (HR) were simultaneously monitored throughout the tests. Rating of perceived exertion (RPE) was assessed immediately after the tests, VO_2_ was also measured for 15 min post-test and peak lactate values were assessed at 7 min post-test. The laboratory conditions were 20–22°C and 38–40% relative humidity. Menstruation was not taken into consideration for female participants, as it has no effect on repeated sprints and anaerobic performance [33].

### Anthropometric and physiological-perceptual measurements

Anthropometric measurements were taken when the participants were fasting (12 h). Height was measured using a portable stadiometer with a 0.1-cm accuracy (Holtain, London, United Kingdom) and body mass and composition were measured with a multifrequency bioelectrical impedance analyzer with a 0.1-kg accuracy (TANITA MC-780, Japan). To measure the participants’ VO_2max_, a mobile cardiopulmonary exercise test device (Cosmed K5, Italy) was used during a ramp procedure on a cycle ergometer [34]. During the Wingate tests, VO_2_ and HR (Polar 810i, Polar Electro, Kempele, Finland) were constantly monitored. Additionally, VO_2_ was measured for 15 min after the tests to observe the fast and slow phases of excess post-exercise oxygen intake (EPOC) and 10 min prior to the tests to identify resting VO_2_ (the last 5 min were used in the analysis). A portable hand analyzer (Lactate Scout +, SensLab GmbH, Germany) was used to quantify lactate from capillary blood samples taken from the left hand’s fingertip before the tests and 1^st^, 3^rd^, 5^th^ and 7^th^ min post-exercise. For each measurement, the manufacturer’s instructions were followed for calibration of the portable metabolic gas analyzer. The Borg’s 15-grade scale (6–20) for RPE was used to assess participant responses, and scores were recorded immediately following each test [35].

### Thirty-second lower-body Wingate tests

Participants completed a typical warm-up consisting of five bouts of 30 seconds each at 100 W (20 s at 60 rpm and 10 s at 110 rpm) on a bike ergometer (894E, Monark, Vansbro, Sweden) before performing the 30-second lower-body Wingate tests with a load set to 0.10 kg·kg^-1^. The test was conducted in both intermittent (IST) and single (SMT) forms. The IST consisted of three 10-second all-out attempts separated by 30 seconds of passive recovery, whereas the SMT was a single 30-second maximal effort. The saddle height was adjusted to the participant’s height to generate 5 to 10 degrees of knee flexion with the foot in the low position of the central void. Participants were instructed to cycle as quickly as possible and were given the same verbal encouragement before the start of each sprint. Performance variables such as peak power (PP), minimum power (MinP) and mean power (MP) were determined by measuring the highest and lowest values throughout the tests. The fatigue index (FI) was determined by calculating the difference between PP and MinP, dividing it by PP and multiplying the result by 100.

### Determination of energy systems contribution

By using breath-by-breath metabolic gas analysis, oxygen uptake during the resting, exercise and 15-min recovery period post-exercise were measured to determine the contributions of the oxidative and ATP-PCr systems. The fast component of EPOC kinetics was used to estimate the ATP-PCr pathway contribution using OriginPro 8.0 software (OriginLab Corp., Northampton, USA). To accurately quantify the ATP-PCr system’s contribution to the energy expended during high-intensity exercise, we employed a mono-exponential model to the EPOC kinetics. This approach involves analyzing the initial rapid increase in oxygen uptake immediately after exercise, which reflects the body’s efforts to replenish the phosphocreatine (PCr) stores depleted during high-intensity activity [36]. The total contribution of the ATP-PCr was calculated using the fast component of EPOC following the final sprint and the sum of VO_2_-time integral during the rest intervals [37, 38]. The contribution from the oxidative pathway was calculated as the exercise-VO_2_ minus resting-VO_2_ [39–41]. Blood samples were taken from the left fingertip before the Wingate test and at 1, 3, 5 and 7 min post-exercise to assess the highest plasma lactate concentration. di Prampero equivalence, in which 1 mmol·L^-1^ of BLa accumulation corresponds to 3 mL of oxygen per kg of body weight, was used to determine the glycolytic contribution [42, 43].

### Statistical analyses

The data were analyzed using SPSS 21.0 (IBM Corp, Armonk, NY, USA), and significance was set at p ≤ 0.05. The data were reported as the mean and standard deviation, and normality was verified using the Shapiro–Wilk test. The independent samples t-test was used to compare the body composition and VO_2max_ test results of male and female groups. Cohen’s d was used to calculate the effect size for the independent samples t-test, and these were classified according to Hopkins [44]. The mixed 2 × 2 (gender × protocol) analysis of variance (ANOVA) with repeated measures was used to compare variables related to different Wingate protocols and gender. Partial eta square values (η_p_^2^) were calculated for the effect size in the ANOVA and effect sizes were classified as small (η_p_^2^≤0.01), medium (0.01<η_p_^2^≤0.06) and large (0.06<η_p_^2^≤0.14).

## Results

Table 1 showed that significant differences in all variables in favor of men, with the exception of age, experience, fat mass, and body mass index. Men had higher absolute VO_2_ (L/min) (p<0.001; d = 2.09), while no significant difference was observed for relative VO_2_ (ml/kg/min) (p = 0.357; d = 0.10).

Table 2 revealed that significant gender-based differences in absolute PP (APP) (F_1;33_ = 40.808; p<0.001; $\eta_{p}^{2}$ = 0.671) and relative PP (RPP) (F_1;33_ = 7.283; p = 0.011; $\eta_{p}^{2}$ = 0.281). Men had significantly higher APP and RPP values than women. However, no significant differences were observed in either protocol effect (APP [F_1;33_ = 0.076; p = 3.508; $\eta_{p}^{2}$ = 0.149], RPP [F_1;33_ = 1.260; p = 0.275; $\eta_{p}^{2}$ = 0.065] as well as gender × protocol interaction (APP [F_1;33_ = 0.011; p = 0.916; $\eta_{p}^{2}$ = 0.001), RPP [F_1;33_ = 0.042; p = 0.840; $\eta_{p}^{2}$ = 0.002]).

**Table 2:** Physiological and performance responses for the 3×10-second IST and 30-second SMT form.

	IST		SMT		Gender effect	Exercise effect	Gender × Exercise Interaction
	Men	Women	Men	Women			
Absolute PP (W)	933.8±143.4	636.8±113.8	965.1±139.3	652.5±105.0	F = 40.808*	F = 3.508	F = 0.011
Relative PP (W.kg^-1^)	12.8±1.8^a^	10.9±1.9	13.2±2.1	11.1±1.5	F = 7.283*	F = 1.260	F = 0.042
Absolute MP (W)	754.9±125.8	530.8±102.6	669.5±7.6	479.6±65.7	F = 38.665*	F = 39.200*	F = 3.965
Relative MP (W.kg^-1^)	10.4±1.6	9.10±1.7	9.1±1.0	8.2±0.9	F = 5.504*	F = 36.371*	F = 0.945
Fatigue index (%)	35.2±6.0	34.6±17.7	56.2±8.6	57.5±9.9	F = 0.000	F = 66.908*	F = 0.052
Lactate delta (mmol.L^-1^)	12.7±2.2	11.7±2.6	10.7±2.9	11.5±2.1	F = 0.021	F = 8.276*	F = 5.647 *
HR peak (bpm)	166.3±10.1	168.0±6.7	170.1±8.3	172.8±9.2	F = 0.008	F = 0.750	F = 0.700
RPE	12.4±1.1	14.2±1.4	15.6±0.5	17.8±0.4	F = 22.412*	F = 40.450*	F = 0.165

IST = intermittent sprint test, SMT = single maximal test, PP = peak power, MP = mean power, HR = heart rate, RPE = rating of perceived exertion.

^a^ *p* < 0.05.

Gender and protocol were found to have significant effects on mean power. Men had higher relative (RPP) and absolute mean power (AMP) than women (AMP [F_1;33_ = 38.665; p<0.001; $\eta_{p}^{2}$ = 0.659] and RPP [F_1;33_ = 5.504; p = 0.029; $\eta_{p}^{2}$ = 0.216]). Furthermore, both women and men achieved higher power values in IST protocol than in SMT protocol (AMP: F_1;33_ = 39.200; p<0.001; $\eta_{p}^{2}$ = 0.662 and RMP [F_1;33_ = 36.371; p<0.001; $\eta_{p}^{2}$ = 0.645]). However, the gender × protocol interaction was not significant (AMP [F_1;33_ = 3.935; p = 0.061; $\eta_{p}^{2}$ = 0.164] and RMP [F_1;33_ = 0.945; p = 0.343; $\eta_{p}^{2}$ = 0.045]).

There was a significant effect of protocol on fatigue index (%), with higher power decrement (PD) observed during SMT protocol compared to IST protocol (F_1;33_ = 66.908; p<0.001; $\eta_{p}^{2}$ = 0.770). There was no gender effect (F_1;33_ = 0.000; p = 0.995; $\eta_{p}^{2}$ = 0.000) or gender × protocol interaction (F_1;33_ = 0.052; p = 0.822; $\eta_{p}^{2}$ = 0.003), indicating no difference in power decrement between men and women for the different repeated sprint protocols.

Significant differences in La_delta_ values were found dependent on the protocol used, with a significant protocol effect (F_1;33_ = 8.276; p = 0.008; $\eta_{p}^{2}$ = 0.294) observed. Gender had no significant effect (F_1;33_ = 0.021; p = 0.885; $\eta_{p}^{2}$ = 0.001) and no interaction effect between gender and protocol was recorded (F_1;33_ = 0.647; p = 0.077; $\eta_{p}^{2}$ = 0.012). Both men and women showed higher La_delta_ responses following IST than SMT.

There was no significant gender effect on the contribution of ATP-PCr (F_1;33_ = 0.137; p = 0.715, $\eta_{p}^{2}$ = 0.006), glycolytic (F_1;33_ = 0.093; p = 0.763; $\eta_{p}^{2}$ = 0.004), and oxidative (F_1;33_ = 0.007; p = 0.933, ƞ^2^ = 0.000) energy pathways (Table 3). However, there was a significant protocol effect on the contribution of ATP-PCr (F_1;33_ = 270.310; p<0.001, $\eta_{p}^{2}$ = 0.928), glycolytic (F_1;33_ = 44,206; p = 0.0001, $\eta_{p}^{2}$ = 0.678) and oxidative (F_1;33_ = 204.552; p<0.001, $\eta_{p}^{2}$ = 0.907) energy pathways. No significant gender × protocol interaction was observed on the contribution of ATP-PCr (F_1;33_ = 0.504; p = 0.612; $\eta_{p}^{2}$ = 0.057), glycolytic (F_1;33_ = 0.074; p = 0.656; $\eta_{p}^{2}$ = 0.004) and oxidative (F_1;33_ = 0.653; p = 0.428; $\eta_{p}^{2}$ = 0.030) energy pathways.

**Table 3:** Estimated relative and absolute energy system contribution during the 3 × 10-second IST and 30-second SMT form.

	IST_sprints_		SMT		Two-way ANOVA		
	Men	Women	Men	Women	Gender effect	Exercise effect	Gender × Exercise Interaction
ATP-PCr (%)	61.4 ± 4.9	63.2 ± 3.4	48.7 ± 6.9	45.45 ± 4.2	F = 0.137	F = 270.310 *	F = 7.504
Glycolytic (%)	31.1 ± 5.7	28.8 ± 5.0	34.5 ± 8.3	38.3 ± 5.5	F = 0.093	F = 44.206 *	F = 0.074
Oxidative (%)	7.6 ± 2.3	8.0 ± 2.6	16.8 ± 2.5	16.2 ± 2.5	F = 0.007	F = 204.552 *	F = 0.653
ATP-PCr (kJ)	105.3 ± 11.6	83.3 ± 8.7	61.4 ± 8.0	45.7 ± 9.1	F = 28.397*	F = 502.519 *	F = 3.009
Glycolytic (kJ)	53.8 ± 13.3	38.8 ± 8.8	44.5 ± 14.1	38.4 ± 8.0	F = 6.036 *	F = 9.153 *	F = 2.172
Oxidative (kJ)	13.2 ± 4.8	10.8 ± 3.7	21.3 ± 3.6	16.2 ± 3.1	F = 8.575*	F = 47.579 *	F = 1.941
Energy Demand (L of O_2_)	8.2 ± 1.0	6.3 ± 0.7	6.1 ± 0.7	4.8 ± 0.8	F = 22.451*	F = 110.874 *	F = 2.451
TEE (kJ)	172.4 ± 21.4	132.9 ± 14.5	127.2 ± 14.5	100.3 ± 16.4	F = 29.070*	F = 131.775*	F = 4.343
PCr__Epocfast_ (L of O_2_)	2.6 ± 0.4	2.2 ± 0.3	2.9 ± 0.4	2.2 ± 0.4	F = 19.003 *	F = 4.275 *	F = 3.211

IST_sprints_ = intermittent sprint test (for sprints only), SMT = single maximal test, TEE = total energy expenditure; PCr__EPOCfast_: = estimated PCr repayment during fast phase of EPOC (kJ).

* *p* < 0.05

**Fig 1:**
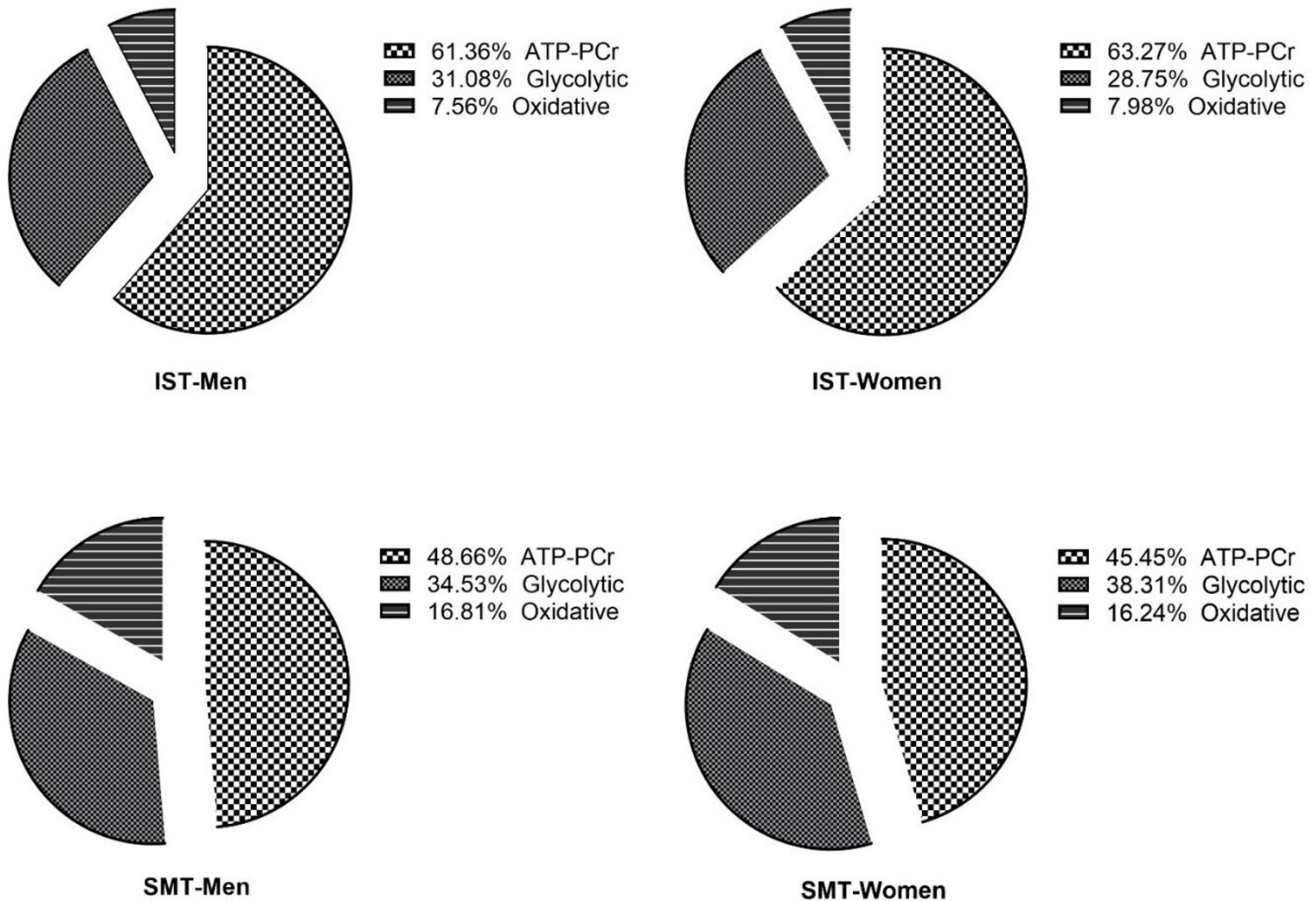
The percentage contribution of energy during IST and SMT protocols (sprints only).

Fig 1 showed that the percent contribution of energy during IST and SMT protocols (sprints only). Significant gender (Total energy expenditure (TEE) [F_1;33_ = 29.070, p = 0.0001, $\eta_{p}^{2}$ = 0.581]; PCr_EPOC_ [F_1;33_ = 19.003; p<0.001; $\eta_{p}^{2}$ = 0.475]) and protocol effects (TEE [F_1;33_ = 131.775; p<0.001; $\eta_{p}^{2}$ = 0.863]; PCr_EPOC_ [F_1;33_ = 4.275; p = 0.050; $\eta_{p}^{2}$ = 0.169]) were observed, with no significant gender × protocol interaction (TEE [F_1;33_ = 4.343; p = 0.060; $\eta_{p}^{2}$ = 0.171]; PCr_EPOC_ [F_1;33_ = 3.211; p = 0.088; $\eta_{p}^{2}$ = 0.133]).

Men and women had higher (TEE) and (PCr_EPOC_) in IST protocol, and men had higher TEE and PCr_EPOC_ than women in both protocols.

## Discussion

To the authors’ current knowledge, this study is the first to compare metabolic energy system contributions, physiological responses and performance outcomes of different forms of Wingate test in combat sports’ athletes according to gender. Our hypothesis that variations in the single all-out and repeated exercise designs would affect the amount of metabolic energy contributed by the bioenergetic pathways overall or during rest periods and would differ between genders was supported by the primary results. Men had higher performance and physiological responses than women, with higher relative and absolute power outputs. However, the fatigue index showed differences according to protocols, whereas no difference was observed between genders.

These results match with those reported in previous researches [21, 45], suggesting that women may have lower anaerobic thresholds than men during repeated sprints leading to a steeper decline in sprint performance at the end of the sprints. A previous study has evidenced that a considerable proportion of type II muscle fibers are activated during maximal cycle sprints [46]. Additionally, smaller cross-sectional areas of type II fibers in women may explain why men usually have higher power values than women in both protocols [46].

Differences in neuromuscular activity between men and women, as well as the reduced mechanical alteration experienced by women during repeated sprint exercises, could be contributing factors to the observed differences in output decrement. It has been shown that women are more efficient at clearing ammonia from the blood than males during repeated 30-second sprints [47]. Additionally, the slight difference in peak power decrement between males and females in both protocols is consistent with prior research [23]. While the performance and physiological variables mentioned produced results that were in line with the literature [26, 48], there is currently a lack of researches comparing the contribution of the metabolic energy systems using various repeated-sprint protocols. Actually, findings related to intermittent maximal exercises within women are not sufficient since the Wingate test is a popular choice for assessing metabolic energy system contributions due to its practical convenience [2, 28, 37, 39, 49].

In both protocols (i.e., IST-SMT), the contribution of the ATP-PCr system was higher. In both groups, ATP-PCr system contribution was greater in IST compared with SMT, whereas the glycolytic and oxidative systems had a higher contribution in SMT. This "lighter" metabolic stress by IST may have allowed for greater PCr resynthesis and lactate removal during the test. The total metabolic energy demand for men and women in IST was 8.2 ± 1.0 and 6.3 ± 0.7 and in SMT 6.1 ± 0.7 and 4.8 ± 0.8 L of O2, respectively, and there was no gender difference in the percentage of metabolic energy system contribution. Outcomes from mechanical variables supported these findings, with higher absolute and relative MP in IST compared with SMT, which had a higher contribution from the TEE and ATP-PCr pathway and, consequently, a lower FI%.

The post-sprint VO_2_-time integral can be used to determine the contribution of PCr stores, given that PCr stores’ recovery is likely to be the main focus of the 30-second rest intervals between sprints [28, 37, 38]. This approach is currently the only non-invasive approach available that can distinguish the contributions of three different metabolic energy systems [2, 50–52]. Researches have demonstrated that VO_2_ during rest intervals can significantly alter metabolic energy pathways’ absolute and percentage contributions during intermittent sprint exercises when the overall exercise duration is matched [26, 36, 53]. The results of this study showed considerable differences between the protocols’ effects on total sprint duration and the percentage of performance decline (Table 3).

The glycolytic system is estimated to provide approximately 45–52% of the lower body’s work during Wingate test [54, 55]. This research determined the glycolytic system’s contribution to a short maximal test (SMT) protocol to be 48.7% for men and 45.5% for women. Studies have reported the aerobic contribution during a lower-body Wingate test ranged from 18–29% [54, 56]. It has been suggested that increasing sprint duration or distance increases the metabolic energy contribution by the aerobic system, but decreases performance [30, 53]. In both groups, increasing relative oxidative system contribution in the SMT resulted in decreased fatigue index (%) and mean power (absolute and relative). Studies have demonstrated that combat athletes possess well-developed anaerobic characteristics [2, 40, 57], and, thus, the relative contribution of the three metabolic energy systems during a 30-second exercise would likely vary between a single maximal exertion and an intermittent sprint. Performance during repeated sprints attempts is largely determined by the balance between phosphocreatine (PCr) storage and resynthesis [28, 30, 53]. PCr storage and resynthesis are key to combat athletes’ success in crucial fight times, as explosive actions rely on ATP-PCr energy contribution. PCr concentrations may drop by as much as 55% after 10 seconds or 83% after 30 seconds of lower-body cycling effort [36, 53], and pH levels may be reduced after additional sprints, which could further inhibit glycolytic enzymes [30, 58].

The majority of PCr (85% of baseline values) and ATP (93% of baseline values) can be resynthesized after a period of passive rest (i.e., 6 min). However, H^+^ concentration may remain significantly higher than baseline values (144 ± 32%) [2, 40, 57]. PCr resynthesis is a complex process, relying heavily on oxygen availability (the fast component) and intramuscular acidosis (the slow component) [30, 58]. Therefore, it is likely that the IST used in the current research caused a minor reduction in PCr compared with SMT. Due to the high glycolytic demand of the Wingate test (used to quantify metabolic energy system contribution [41, 49, 56], it is speculated that shorter work bouts (10s "all-out") or increased resistance during training may be necessary to elicit performance improvements (peak and mean power and total work [28, 36].

Longer-term training programs with higher work-to-rest ratios (e.g., 1:30, 1:36 and 1:42) are needed to induce changes in the use of oxidative and glycolytic energy systems. The rate of phosphocreatine resynthesis is affected by various factors, such as muscle pH and adenosine diphosphate concentrations, as highlighted in a review by McMahon and Jenkins [59]. This is supported by a previous study [29], which found that musculoskeletal performance depends on non-mitochondrial metabolism for mechanical function during sprints, regardless of the absolute force produced. Additionally, a strong correlation was found between the results of the Wingate test and the contributions of the phosphagen and glycolytic pathways measured by maximal accumulated oxygen deficit (PP and ATP-PCr) [60]. The importance of the PCr has been highlighted as a determinant for ATP resynthesis during RSA (10 × 6 s sprints with 30 s of passive recovery on a cycle ergometer) [28] and 12 × 20 m running sprints with 20 s of recovery [61]. This emphasizes the significance of non-mitochondrial metabolism in force/power generation during all-out efforts, particularly the ATP-PCr, despite some differences between the test protocols of the current research and the studies previously cited [61].

In both groups, PCr_EPOCfast_ was higher in SMT than IST. This may be due to variations in exercise intensity, since it is known that exercise intensity and EPOC magnitude and duration are correlated [62–64]. Men had higher absolute and relative power output on each Wingate bout compared to women, with high effect sizes, indicating that exercise intensity was also higher. However, no differences in HR peak and lactate, which can also be used as indicators of exercise intensity, were observed, despite being affected by sex- and hormone-related differences [65, 66]. Compared with previous studies [50, 67] that used the same methodology to assess metabolic energy demand and energy system contributions in repeated exercises, our study produced higher energy demands (IST: 172.4–132.9 kJ·min^-1^; SMT: 127.2–100.3 kJ·min^-1^ for men and women, respectively) due to the inclusion of sprint exercises, which caused more metabolic stress in both groups (Table 3). The improvement and testing of anaerobic systems (ATP-PCr and glycolytic systems), which are thought to be essential metabolic factors for the performance of repeated-sprint exercises, were also compared across various designs in this research. Exercises performed repeatedly at maximum effort with brief rest periods have the benefit of increasing the percentage of anaerobic energy expenditure, particularly that from the phosphagen system.

This study has some limitations in calculating the metabolic energy contribution from the glycolytic and phosphagen systems. For example, lactate concentrations were only measured before and after the protocols, not during the exercises, thus not providing data on lactate kinetics during exercise. Additionally, the oxygen consumed during the recovery intervals and during the fast phase of EPOC was entirely attributed to the ATP-PCr system, disregarding the amount of oxygen rebinding to myoglobin. Though the current approach provided a theoretical estimation of the distinct fraction of energy metabolism, more precise data could not be obtained regarding physiological processes at the cellular level.

## Conclusions

This study provides critical insights into the contributions of metabolic energy systems during the 30-second Wingate anaerobic test among combat sports athletes. By comparing the IST and SMT forms of the test and examining gender differences, the research contributes significantly to our understanding of energy system dynamics in high-intensity exercise. The findings suggest that TEE and ATP-PCr system contribution are higher in IST than in SMT, offering valuable implications for training strategies in combat sports. Although both energy systems contribute to performance, the differential impact observed between exercise forms underscores the importance of tailored training programs. Furthermore, the absence of significant exercise form and gender interaction highlights the universal applicability of these results across genders, providing a foundational basis for optimizing athlete performance in combat sports. Its strength lies in the depth of analysis, robust methodology, and the practical relevance of its findings, which offer insights for optimizing training strategies in combat sports. However, the study’s scope, centered on highly trained athletes, may limit the applicability of its results to wider populations. Future research is encouraged to broaden participant diversity, incorporate longitudinal training studies, and utilize advanced measurement techniques for a more detailed physiological understanding. Overall, this investigation highlights the significance of tailored training programs, acknowledging the distinct metabolic demands and individual athlete characteristics, thereby contributing to the enhancement of athletic performance and efficiency in combat sports. In conclusion, the study’s exploration of energy system contributions during high-intensity exercise offers essential contributions to sports science, particularly within the realm of combat sports, paving the way for future research to build upon these findings and explore practical applications further.

## References

[1] Franchini E. Energy System Contributions during Olympic Combat Sports: A Narrative Review. Metabolites. 2023;13(2):297 doi: 10.3390/metabo13020297 36837916 PMC9961508

[2] Ulupınar S, Özbay S. Energy pathway contributions during 60-second upper-body Wingate test in Greco-Roman wrestlers: intermittent versus single forms. Research in Sports Medicine. 2022;30(3):244–255 doi: 10.1080/15438627.2021.1895784 33663306

[3] Franchini E, Herrera-Valenzuela T. Special issue—strength and conditioning for combat sports athletes. Rev Art Marc Asi. 2021;16:1–203

[4] Pessôa Filho DM, Sancassani A, da Cruz Siqueira LO, Massini DA, Almeida Santos LG, Neiva CM, et al. Energetics contribution during no-gi Brazilian jiu jitsu sparring and its association with regional body composition. Plos one. 2021;16(11):e0259027 doi: 10.1371/journal.pone.0259027 34767563 PMC8589206

[5] Franchini E, Herrera-Valenzuela T. Strength and conditioning for combat sports athletes. Revista de Artes Marciales Asiáticas. 2021;16(1s):1–203

[6] Wang X, Soh KG, Samsudin S, Deng N, Liu X, Zhao Y, Akbar S. Effects of high-intensity functional training on physical fitness and sport-specific performance among the athletes: A systematic review with meta-analysis. Plos one. 2023;18(12):e0295531 doi: 10.1371/journal.pone.0295531 38064433 PMC10707569

[7] Bruzas V, Venckunas T, Kamandulis S, Snieckus A, Mockus P, Stasiulis A. Metabolic and physiological demands of 3× 3-min-round boxing fights in highly trained amateur boxers. The Journal of Sports Medicine and Physical Fitness. 2022;63(5):623–629 35415997 10.23736/S0022-4707.22.13661-3

[8] Ojeda-Aravena A, Herrera-Valenzuela T, Valdés-Badilla P, Martín B-S, Thapa RK, Ramirez-Campillo R. A Systematic Review with Meta-Analysis on the Effects of Plyometric-Jump Training on the Physical Fitness of Combat Sport Athletes. Sports. 2023;11(2):33 doi: 10.3390/sports11020033 36828318 PMC9965890

[9] Franchini E. High-intensity interval training prescription for combat-sport athletes. International journal of sports physiology and performance. 2020;15(6):767–776 doi: 10.1123/ijspp.2020-0289 32502972

[10] Sloth M, Sloth D, Overgaard K, Dalgas U. Effects of sprint interval training on VO 2max and aerobic exercise performance: a systematic review and meta‐analysis. Scandinavian journal of medicine & science in sports. 2013;23(6):e341–e352 23889316 10.1111/sms.12092

[11] Gillen JB, Percival ME, Skelly LE, Martin BJ, Tan RB, Tarnopolsky MA, et al. Three minutes of all-out intermittent exercise per week increases skeletal muscle oxidative capacity and improves cardiometabolic health. PloS one. 2014;9(11):e111489 doi: 10.1371/journal.pone.0111489 25365337 PMC4218754

[12] Burgomaster KA, Heigenhauser GJ, Gibala MJ. Effect of short-term sprint interval training on human skeletal muscle carbohydrate metabolism during exercise and time-trial performance. Journal of applied physiology. 2006;100(6):2041–2047 doi: 10.1152/japplphysiol.01220.2005 16469933

[13] Spencer M, Dawson B, Goodman C, Dascombe B, Bishop D. Performance and metabolism in repeated sprint exercise: effect of recovery intensity. European journal of applied physiology. 2008;103(5):545–552 doi: 10.1007/s00421-008-0749-z 18443815

[14] Peyrebrune M, Toubekis A, Lakomy H, Nevill M. Estimating the energy contribution during single and repeated sprint swimming. Scandinavian journal of medicine & science in sports. 2014;24(2):369–376 doi: 10.1111/j.1600-0838.2012.01517.x 22897515

[15] Ball D, Burrows C, Sargeant AJ. Human power output during repeated sprint cycle exercise: the influence of thermal stress. European journal of applied physiology and occupational physiology. 1999;79:360–366 doi: 10.1007/s004210050521 10090637

[16] Padulo J, Ardigò L, Attene G, Cava C, Wong D, Chamari K, et al. The effect of slope on repeated sprint ability in young soccer players. Research in Sports Medicine. 2016;24(4):320–330 doi: 10.1080/15438627.2016.1222276 27537203

[17] Padulo J, Tabben M, Attene G, Ardigò L, Dhahbi W, Chamari K. The impact of jumping during recovery on repeated sprint ability in young soccer players. Research in Sports Medicine. 2015;23(3):240–252 doi: 10.1080/15438627.2015.1040919 26038845

[18] Ulupınar S, Özbay S, Gençoğlu C, Franchini E, Kishalı NF, Ince I. Effects of sprint distance and repetition number on energy system contributions in soccer players. Journal of Exercise Science & Fitness. 2021;19(3):182–188 doi: 10.1016/j.jesf.2021.03.003 33889186 PMC8044429

[19] Hunter SK. Sex differences in human fatigability: mechanisms and insight to physiological responses. Acta physiologica. 2014;210(4):768–789 doi: 10.1111/apha.12234 24433272 PMC4111134

[20] Franchini E, Cormack S, Takito MY. Effects of high-intensity interval training on olympic combat sports athletes’ performance and physiological adaptation: A systematic review. The Journal of Strength & Conditioning Research. 2019;33(1):242–252 doi: 10.1519/JSC.0000000000002957 30431531

[21] Billaut F, Smith K. Sex alters impact of repeated bouts of sprint exercise on neuromuscular activity in trained athletes. Applied Physiology, Nutrition, and Metabolism. 2009;34(4):689–699 doi: 10.1139/H09-058 19767805

[22] Bishop DJ. Fatigue during intermittent‐sprint exercise. Clinical and Experimental Pharmacology and Physiology. 2012;39(9):836–841 doi: 10.1111/j.1440-1681.2012.05735.x 22765227

[23] Soydan TA, Hazir T, Ozkan A, Kin-Isler A. Gender differences in repeated sprint ability. Isokinetics and Exercise Science. 2018;26(1):73–80

[24] Henderson GC, Fattor JA, Horning MA, Faghihnia N, Johnson ML, Mau TL, et al. Lipolysis and fatty acid metabolism in men and women during the postexercise recovery period. The Journal of physiology. 2007;584(3):963–981 doi: 10.1113/jphysiol.2007.137331 17855762 PMC2277001

[25] Oosthuyse T, Bosch AN. Oestrogen’s regulation of fat metabolism during exercise and gender specific effects. Current opinion in pharmacology. 2012;12(3):363–371 doi: 10.1016/j.coph.2012.02.008 22398320

[26] Milioni F, Zagatto AM, Barbieri RA, Andrade VL, dos Santos JW, Gobatto CA, et al. Energy systems contribution in the running-based anaerobic sprint test. International journal of sports medicine. 2017;38(03):226–232 doi: 10.1055/s-0042-117722 28192833

[27] Breenfeldt Andersen A, Bejder J, Bonne T, Olsen NV, Nordsborg N. Repeated Wingate sprints is a feasible high-quality training strategy in moderate hypoxia. PLoS One. 2020;15(11):e0242439 doi: 10.1371/journal.pone.0242439 33186393 PMC7665825

[28] Gaitanos, Williams C, Boobis LH, Brooks S. Human muscle metabolism during intermittent maximal exercise. Journal of applied physiology. 1993;75(2):712–719 doi: 10.1152/jappl.1993.75.2.712 8226473

[29] Mendez-Villanueva A, Hamer P, Bishop D. Fatigue in repeated-sprint exercise is related to muscle power factors and reduced neuromuscular activity. European journal of applied physiology. 2008;103:411–419 doi: 10.1007/s00421-008-0723-9 18368419

[30] Bogdanis GC, Nevill ME, Boobis LH, Lakomy H. Contribution of phosphocreatine and aerobic metabolism to energy supply during repeated sprint exercise. Journal of applied physiology. 1996;80(3):876–884 doi: 10.1152/jappl.1996.80.3.876 8964751

[31] Bogdanis G, Nevill M, Lakomy H, Boobis L. Power output and muscle metabolism during and following recovery from 10 and 20 s of maximal sprint exercise in humans. Acta Physiologica Scandinavica. 1998;163(3):261–272 doi: 10.1046/j.1365-201x.1998.00378.x 9715738

[32] Cherry P, Lakomy H, Boobis L, Nevill M. Rapid recovery of power output in females. Acta physiologica scandinavica. 1998;164(1):79–87 doi: 10.1046/j.1365-201X.1998.00397.x 9777028

[33] Wiecek M, Szymura J, Maciejczyk M, Cempla J, Szygula Z. Effect of sex and menstrual cycle in women on starting speed, anaerobic endurance and muscle power. Acta Physiologica Hungarica. 2016;103(1):127–132 doi: 10.1556/036.103.2016.1.13 27030635

[34] Basset FA, Boulay MR. Specificity of treadmill and cycle ergometer tests in triathletes, runners and cyclists. European Journal of Applied Physiology. 2000;81:214–221 doi: 10.1007/s004210050033 10638380

[35] Borg GA. Psychophysical bases of perceived exertion. Medicine & science in sports & exercise. 1982 7154893

[36] Gastin PB. Energy system interaction and relative contribution during maximal exercise. Sports medicine. 2001;31:725–741 doi: 10.2165/00007256-200131100-00003 11547894

[37] Bogdanis, Nevill ME, Boobis LH, Lakomy H, Nevill AM. Recovery of power output and muscle metabolites following 30 s of maximal sprint cycling in man. The Journal of physiology. 1995;482(2):467–480 doi: 10.1113/jphysiol.1995.sp020533 7714837 PMC1157744

[38] Dawson B, Goodman C, Lawrence S, Preen D, Polglaze T, Fitzsimons M, et al. Muscle phosphocreatine repletion following single and repeated short sprint efforts. Scandinavian journal of medicine & science in sports. 1997;7(4):206–213 doi: 10.1111/j.1600-0838.1997.tb00141.x 9241025

[39] La Monica MB, Fukuda DH, Starling-Smith TM, Clark NW, Panissa VL. Alterations in energy system contribution following upper body sprint interval training. European journal of applied physiology. 2020;120:643–651 doi: 10.1007/s00421-020-04304-w 31974857

[40] Julio UF, Panissa VL, Esteves JV, Cury RL, Agostinho MF, Franchini E. Energy-system contributions to simulated judo matches. International Journal of Sports Physiology and Performance. 2017;12(5):676–683 doi: 10.1123/ijspp.2015-0750 27736247

[41] Julio UF, Panissa VL, Cury RL, Agostinho MF, Esteves JV, Franchini E. Energy system contributions in upper and lower body wingate tests in highly trained athletes. Research quarterly for exercise and sport. 2019;90(2):244–250 doi: 10.1080/02701367.2019.1576839 30908121

[42] Spencer M, Bishop D, Dawson B, Goodman C. Physiological and metabolic responses of repeated-sprint activities: specific to field-based team sports. Sports medicine. 2005;35:1025–1044 doi: 10.2165/00007256-200535120-00003 16336007

[43] di Prampero PE, Ferretti G. The energetics of anaerobic muscle metabolism: a reappraisal of older and recent concepts. Respiration physiology. 1999;118(2–3):103–115 doi: 10.1016/s0034-5687(99)00083-3 10647856

[44] Hopkins W, Marshall S, Batterham A, Hanin J. Progressive statistics for studies in sports medicine and exercise science. Medicine+ Science in Sports+ Exercise. 2009;41(1):3 doi: 10.1249/MSS.0b013e31818cb278 19092709

[45] Billaut F, Giacomoni M, Falgairette G. Maximal intermittent cycling exercise: effects of recovery duration and gender. Journal of Applied Physiology. 2003;95(4):1632–1637 doi: 10.1152/japplphysiol.00983.2002 12794037

[46] Casey A, Constantin-Teodosiu D, Howell S, Hultman E, Greenhaff P. Metabolic response of type I and II muscle fibers during repeated bouts of maximal exercise in humans. American Journal of Physiology-Endocrinology and Metabolism. 1996;271(1):E38–E43 doi: 10.1152/ajpendo.1996.271.1.E38 8760079

[47] Esbjornsson M, Bulow J, Norman B, Simonsen L, Nowak J, Rooyackers O, et al. Adipose tissue extracts plasma ammonia after sprint exercise in women and men. Journal of applied physiology. 2006;101(6):1576–1580 doi: 10.1152/japplphysiol.01119.2004 16282425

[48] Little T, Williams AG. Effects of sprint duration and exercise: rest ratio on repeated sprint performance and physiological responses in professional soccer players. The Journal of Strength & Conditioning Research. 2007;21(2):646–648 doi: 10.1519/R-20125.1 17530972

[49] Franchini E, Takito MY, Kiss MAPDM. Performance and energy systems contributions during upper-body sprint interval exercise. Journal of exercise rehabilitation. 2016;12(6):535 doi: 10.12965/jer.1632786.393 28119874 PMC5227314

[50] Panissa VL, Fukuda DH, Caldeira RS, Gerosa-Neto J, Lira FS, Zagatto AM, et al. Is oxygen uptake measurement enough to estimate energy expenditure during high-intensity intermittent exercise? Quantification of anaerobic contribution by different methods. Frontiers in physiology. 2018;9:868 doi: 10.3389/fphys.2018.00868 30038583 PMC6046462

[51] Davis P, Leithäuser RM, Beneke R. The energetics of semicontact 3× 2-min amateur boxing. International journal of sports physiology and performance. 2014;9(2):233–23924572964

[52] Franchini E. Response to Beneke and Hoos: Letters to the Editor. International journal of sports physiology and performance. 2012;7(4):308–309 23281504

[53] Girard O, Mendez-Villanueva A, Bishop D. Repeated-sprint ability—part I: factors contributing to fatigue. Sports medicine. 2011;41:673–694 doi: 10.2165/11590550-000000000-00000 21780851

[54] Bogdanis G, Nevill M, Lakomy H. Effects of previous dynamic arm exercise on power output during repeated maximal sprint cycling. Journal of sports sciences. 1994;12(4):363–370 doi: 10.1080/02640419408732182 7932946

[55] Beneke R, Hütler M, Leithäuser RM. Anaerobic performance and metabolism in boys and male adolescents. European journal of applied physiology. 2007;101:671–677 doi: 10.1007/s00421-007-0546-0 17710431

[56] Beneke R, Pollmann C, Bleif I, Leithäuser R, Hütler M. How anaerobic is the Wingate Anaerobic Test for humans? European journal of applied physiology. 2002;87:388–392 doi: 10.1007/s00421-002-0622-4 12172878

[57] Slimani M, Chaabene H, Miarka B, Franchini E, Chamari K, Cheour F. Kickboxing review: anthropometric, psychophysiological and activity profiles and injury epidemiology. Biology of sport. 2017;34(2):185–196 doi: 10.5114/biolsport.2017.65338 28566813 PMC5424459

[58] Mendez-Villanueva A, Edge J, Suriano R, Hamer P, Bishop D. The recovery of repeated-sprint exercise is associated with PCr resynthesis, while muscle pH and EMG amplitude remain depressed. PloS one. 2012;7(12):e51977 doi: 10.1371/journal.pone.0051977 23284836 PMC3524088

[59] McMahon S, Jenkins D. Factors affecting the rate of phosphocreatine resynthesis following intense exercise. Sports Medicine. 2002;32:761–784 doi: 10.2165/00007256-200232120-00002 12238940

[60] Bertuzzi R, Kiss M, Damasceno M, Oliveira R, Lima-Silva A. Association between anaerobic components of the maximal accumulated oxygen deficit and 30-second Wingate test. Brazilian Journal of Medical and Biological Research. 2015;48:261–266 doi: 10.1590/1414-431X20144043 25627804 PMC4381947

[61] Wadley G, Le Rossignol P. The relationship between repeated sprint ability and the aerobic and anaerobic energy systems. Journal of Science and Medicine in Sport. 1998;1(2):100–110 doi: 10.1016/s1440-2440(98)80018-2 9732114

[62] Laforgia J, Withers RT, Gore CJ. Effects of exercise intensity and duration on the excess post-exercise oxygen consumption. Journal of sports sciences. 2006;24(12):1247–1264 doi: 10.1080/02640410600552064 17101527

[63] McGarvey W, Jones R, Petersen S. Excess post-exercise oxygen consumption following continuous and interval cycling exercise. International journal of sport nutrition and exercise metabolism. 2005;15(1):28–37 doi: 10.1123/ijsnem.15.1.28 15902987

[64] Williams CB, Zelt JG, Castellani LN, Little JP, Jung ME, Wright DC, et al. Changes in mechanisms proposed to mediate fat loss following an acute bout of high-intensity interval and endurance exercise. Applied Physiology, Nutrition, and Metabolism. 2013;38(12):1236–1244 doi: 10.1139/apnm-2013-0101 24195624

[65] Forsyth JJ, Reilly T. The combined effect of time of day and menstrual cycle on lactate threshold. Medicine & Science in Sports & Exercise. 2005;37(12):2046–2053 doi: 10.1249/01.mss.0000179094.47765.d0 16331128

[66] Pivarnik JM, Marichal CJ, Spillman T, Morrow J Jr. Menstrual cycle phase affects temperature regulation during endurance exercise. Journal of Applied Physiology. 1992;72(2):543–548 doi: 10.1152/jappl.1992.72.2.543 1559930

[67] Latzel R, Hoos O, Stier S, Kaufmann S, Fresz V, Reim D, Beneke R. Energetic profile of the basketball exercise simulation test in junior elite players. International Journal of Sports Physiology and Performance. 2018;13(6):810–815 doi: 10.1123/ijspp.2017-0174 29182413

