# Correction: The contribution of energy systems during 30-second lower body Wingate anaerobic test in combat sports athletes: Intermittent versus single forms and gender comparison

**DOI:** 10.1371/journal.pone.0306361

**Published:** 2024-06-27

**Authors:** The PLOS ONE Editors

## Notice of Republication

This article [1] was republished on June 4th, 2024, to address an issue identified post-publication. An updated version of S1 File is provided with this notice. Please download this article again to view the correct version.

The article’s Data Availability statement is updated to: All relevant data are within the paper and its Supporting Information file.

## Supporting information

S1 File Underlying dataset. A) Descriptive characteristics of athletes including age range, height, weight; B) Wingate test outputs, including metrics such as peak power, mean power, and fatigue index; C) Oxygen Consumption (O2) Data for two different Wingate test protocols, providing comparative insights into athletes’ oxygen consumption during these tests.
(ZIP)
